# Medical treatment of fibroids: FIGO best practice guidance

**DOI:** 10.1002/ijgo.70497

**Published:** 2025-09-10

**Authors:** Ivonne Diaz, Mary Ann Lumsden, Magdalena Zeppernick

**Affiliations:** ^1^ Fertility Unit PMA Sanitas University and Nueva Granada Military University Medical School Bogotá Colombia; ^2^ School of Medicine University of Glasgow Glasgow UK; ^3^ Department of Gynecology and Obstetrics Justus Liebig University Giessen Giessen Germany

**Keywords:** combined oral contraceptives, fibroids, gonadotropin‐releasing hormone analogs, progesterone receptor modulators, progestins, tranexamic acid

## Abstract

Even though uterine fibroids are a widespread condition, the range of approved medical treatment options remains limited. In fact, only a few drugs are officially approved for the therapy of fibroids. In both the USA and the European Medicines Agency region, selected gonadotropin‐releasing hormone (GnRH) antagonists have been approved for this indication. GnRH analogs are primarily indicated for the preoperative management of uterine fibroids, largely because of their unfavorable long‐term adverse effect profile. The previously approved therapy with ulipristal acetate had to undergo extensive restrictions because of safety concerns. Nevertheless, there are additional medications that may be used in clinical practice. These include combined oral contraceptives, oral progestins, the levonorgestrel‐releasing intrauterine system (LNG‐IUS), and tranexamic acid. Although not specifically approved for the treatment of fibroids, these agents can reduce the intensity of bleeding in cases of heavy menstrual bleeding and thereby improve symptoms and lead to an improved quality of life for the patients.

## 1 INTRODUCTION

There are different options for treating uterine fibroids. In many cases, medical treatment is the first‐line treatment to preserve fertility and avoid surgery,^1^ and is a perfectly satisfactory solution for the patient. Beyond this, medical treatment can be used presurgically to minimize intraoperative complications by reducing the volume of the fibroid(s) or increasing the level of hemoglobin before surgery is performed.

Other treatment options in symptomatic women include interventional radiology and surgical procedures,^2^ and these will be presented in other papers in this collection.

Before one can decide which preparation should be used, it is obligatory to decide whether a treatment is necessary at all. Most women with uterine fibroids are asymptomatic and therefore require no intervention. Approximately 30% of them will present with symptoms, which can include abnormal uterine bleeding, anemia, pelvic pain and pressure, back pain, urinary frequency, constipation, or infertility, and will require intervention.^1^ The treatment options depend on the personal treatment objectives of the patients in addition to expected treatment effectiveness. Each therapy has its own advantages and disadvantages and improves fibroid volume, pain, menstrual bleeding, and/or anemia^3^ in different ways (Table 1).

**TABLE 1:** Medical treatment options for uterine fibroids.

Type	Option
*Non‐hormonal*	
	Tranexamic acid
*Hormonal*	
	Progestins Levonorgestrel‐releasing intrauterine systems Oral progestins
	Combined oral contraceptives
	Gonadotropin‐releasing hormone analogs Gonadotropin‐releasing hormone agonists Gonadotropin‐releasing hormone antagonists
	Progesterone‐receptor modulators

## 2 MEDICAL TREATMENT OPTIONS

### 2.1 Tranexamic acid

Tranexamic acid (TXA) is a suitable option for the management of abnormal uterine bleeding associated with uterine fibroids. TXA is a synthetic lysine‐analog antifibrinolytic^4^ and therefore not a causal therapy for uterine fibroids. By inhibiting fibrinolysis, it helps to manage the bleeding but does not affect the size of the fibroids.

TXA has shown an average reduction of 89 mL in menstrual bleeding in patients with uterine fibroids.^5^ In patients with heavy menstrual bleeding, a 40%–50% reduction in bleeding has been described.^6^ TXA appears effective for treating heavy menstrual bleeding compared with placebo, non‐steroidal anti‐inflammatory drugs, oral luteal progestogens, ethamsylate, or herbal remedies, but may be less effective than levonorgestrel intrauterine systems.^6^ Intravenous infusion of TXA in the intraoperative period of large myomectomies showed no significant difference compared with placebo.^7^, ^8^

TXA can be administered intravenously or orally during menstruation.^9^

Adverse events associated with TXA are gastrointestinal adverse effects, abdominal discomfort, headaches, dizziness, breast tenderness, dysmenorrhea, weight changes, and mood swings. No thromboembolic events have been reported with TXA use.^6^ The contraindications, apart from a known allergy to TXA, are history of venous or arterial thromboembolism or active thromboembolic disease.^9^

### 2.2 Progestins

#### 2.2.1 Local—Levonorgestrel‐releasing intrauterine system

The levonorgestrel‐releasing intrauterine system (LNG‐IUS) is a T‐shaped device that releases levonorgestrel (a synthetic derivative of the hormone progesterone) into the uterus—originally to prevent pregnancy. In 2009, the US Food and Drug Administration (FDA) also approved this intrauterine device for additional use to treat heavy menstrual bleeding.^10^

A prospective comparative study investigated the effect of LNG‐IUS use in women with fibroid‐related heavy menstrual bleeding.^11^ Within 1 month of LNG‐IUS insertion, the Pictorial Blood Loss Assessment Chart score fell by 86.8% (*P* < 0.0001). At 3, 12, 24, 36, and 48 months, the menstrual blood loss was reduced by 92.1%, 97.4%, 97.4%, 99.5%, and 99.5%, respectively. The mean uterine volume was significantly reduced, but there was no statistically significant reduction in the fibroid volume (*P* = 0.409). These findings, along with additional information such as the increase in blood hemoglobin and ferritin levels following treatment, have been confirmed in other studies.^12^, ^13^

Various types of LNG‐IUS are available, offering durations of action between 3 and 5 years. Patients should be informed that there is a risk of device expulsion, which has been associated with uterine fibroids larger than 3 cm in diameter, though not with the location of the fibroids.

#### 2.2.2 Systemic—Oral progestins

Oral progestins have different effects that may be interesting for fibroid‐associated problems. Progestins are not only able to stabilize endometrial fragility and inhibit endometrial growth but also inhibit angiogenesis. These steroids stimulate the conversion of estradiol into the less active estrone. They prevent ovulation and ovarian steroidogenesis, interrupting the production of estrogen receptors and the estrogen‐dependent stimulation of the endometrium, leading to an atrophic endometrium.^14^ In summary, there are various bleeding‐reducing effects. Bleeding control has been confirmed in different contexts.

In women with ovulatory abnormal uterine bleeding, oral medroxyprogesterone acetate (MPA), norethindrone, megestrol acetate, or micronized progesterone taken cyclically (starting on menstrual day 5 for 21 days) or continuously provides cycle control and reduction of menstrual blood loss.^14^ Norethisterone decreases the volume of uterine bleeding determined by Visual Bleeding Score by 56% over 6 months of treatment.^15^

In patients presenting with acute abnormal uterine bleeding, a multidose progestin (e.g. MPA 20 mg, three times daily for 1 week, followed by daily dosing for 3 weeks) can significantly reduce menstrual blood loss.^16^

A systematic review published in the Cochrane Library compared progestogens with progestogen‐releasing intrauterine systems for uterine fibroids. The review reported spotting as the adverse event more likely to be reported by women with the LNG‐IUS (64.3%) than by those taking norethisterone acetate (30%).^17^ Based on the data reviewed, the authors concluded that there is only low‐quality evidence and the superiority of oral progestogens remains uncertain. Consequently, the use of oral progestins for the treatment of uterine fibroids is not strongly supported by current evidence and should only be considered in select cases.

Oral progestins may be a good option for women whose primary symptom is heavy menstrual bleeding and who also require contraception. In these cases, the oral progestins have multiple benefits. Compared with other medical options they are cost‐effective, severe adverse effects are limited, and they are approved for contraception. However, it is important to keep in mind that oral progestins do not reduce fibroid volume.

### 2.3 Estrogen‐progestin contraceptives

Combined oral contraceptives (COCs) are composed of an estrogen and a progestin in a single tablet for preventing pregnancy. Today, these agents offer a broader range of effects and are used in the treatment of multiple indications including uterine fibroid therapy.^18^

COCs reduce abnormal uterine bleeding in women with uterine fibroids by 9.9 mL (13.4% reduction) as measured by the alkaline hematin method, and by 53.5% by the Pictorial Blood Loss Assessment Chart.^17^ A meta‐analysis of studies assessing the association of COCs with fibroid growth demonstrated a 17% reduction in the risk of growth in current users, although the authors pointed out significant heterogeneity among the included trials.^19^ There is no evidence to suggest that COCs promote the growth of uterine fibroids.^20^

The available data indicate that COCs may be a treatment option for women experiencing heavy menstrual bleeding associated with uterine fibroids. However, there is limited evidence regarding their efficacy for other fibroid‐related symptoms, particularly as COCs do not significantly reduce fibroid volume. Another important factor to consider is the patient's age. Uterine fibroids often grow with advancing age, meaning that symptoms frequently manifest during later reproductive years. The use of COCs in older women remains a subject of debate. In such cases, it is advisable to consider formulations with lower estrogen doses and carefully selected progestogens to minimize the risk of thrombotic or cardiovascular events. Moreover, women over the age of 35 years who smoke should not use COCs due to an increased risk of serious complications.^21^ In summary, COCs cannot be considered a universal treatment strategy for uterine fibroids, particularly in older patients.

A review of the literature evaluated the impact of hormonal replacement treatment (HRT) in postmenopausal women with uterine fibroids.^20^ Available data suggest that uterine fibroids might be influenced by HRT, without representing an absolute contraindication. Women with uterine fibroids who use HRT should be periodically examined and hormonal treatment should be discontinued if fibroids appear to increase in size.

### 2.4 Gonadotropin‐releasing hormone analogs

#### 2.4.1 Gonadotropin‐releasing hormone agonists

Gonadotropin‐releasing hormone (GnRH) agonists are peptides similar in structure to GnRH with amino acid substitutions that enhance their affinity for binding to the receptor. This causes continuous stimulation, initially producing an increase in the release of gonadotropins (flare effect) that can last 1–2 weeks, after which gonadal sex steroids decrease until reaching medical castration levels.^22^

GnRH agonists are effective for shrinking fibroid size (about 50% of their initial volume). Several randomized, placebo‐controlled studies showed that leuprolide acetate significantly reduces uterine and fibroid volume. This results in improvement of fibroid‐related symptoms. Unfortunately, this highly effective therapy is limited in duration because of its adverse effects. It is restricted to a 3‐ to 6‐month interval, and when treatment is stopped, the fibroids come back to their pretreatment size.^10^ Consequently, therapy with GnRH analogs is not suitable as a long‐term treatment—although it can offer significant benefits in the preoperative setting—unless given with “add‐back” hormones, which at low doses do not affect the success of the treatment with regard to symptoms but do minimize long‐term adverse effects.^23^

In reference to the use of GNRH analogs before surgery, the preoperative use of GnRH agonists is associated with an increase in preoperative hemoglobin, and a reduction in both uterine and fibroid volume. These effects come at the expense of a greater likelihood (odds) of adverse events, in particular hot flushes, headache, dizziness, vaginitis, change in breast size, and sweating.^24^ A Cochrane systematic review published in 2017 showed these principal outcomes during and after the hysterectomy. Other benefits included: a significant reduction in blood loss and the need for blood transfusions, smaller odds of requiring vertical incision, lower likelihood (odds) of difficult surgery and improved postoperative hemoglobin levels. In women undergoing myomectomy, most trials found that GnRH agonists reduced intraoperative blood loss, there was no evidence that pretreatment influenced duration of surgery, odds of intraoperative blood transfusions, duration of hospital stay, and recurrence of fibroids or postoperative hemoglobin levels.^24^

A meta‐analysis published in 2020 that included 213 women undergoing myomectomy by hysteroscopy showed no statistically significant difference in the use of GnRH agonists compared with the control group for complete resection of submucosal fibroids (relative risk [RR] 0.94, 95% confidence interval [CI] 0.80–1.11); operative time (mean difference [MD] –3.81, 95% CI –3.81 to 2.13); fluid absorption (MD –65.90, 95% CI –9.75 to 2.13); or complications (RR 0.92, 95% CI 0.18–4.82) so the authors do not recommend routine preoperative use of GnRH agonists in these cases.^25^

The resulting hypoestrogenism can cause complications such as hot flushes, vaginal dryness, bone mass affectation, among others. For this reason, GnRH analogs should not be used for a prolonged period of time.^26^ As an alternative, an adjuvant substitutive treatment or add‐back strategy can be applied. A Cochrane study showed that tibolone, raloxifene, estriol, and ipriflavone help to preserve bone density and that MPA and tibolone can reduce vasomotor symptoms. Some drugs used, such as MPA, tibolone, and conjugated estrogens, showed an increase in uterine volume as an adverse event.^27^

#### 2.4.2 Gonadotropin‐releasing hormone antagonists

Knowing the pathophysiology of fibroid growth, a possibility of abolishing GnRH stimulation becomes an alternative medical treatment. GnRH antagonists bind reversibly to GnRH receptors without stimulating them, so they do not produce a flare‐up effect, producing medical castration levels after 24–72 hours following drug administration.^22^

A systematic review and meta‐analysis evaluated the use of GnRH antagonists for oral administration.^28^ They showed that elagolix at doses of 600 mg, 400 mg, and 200 mg is effective in suppressing bleeding, with a greater likelihood of amenorrhea at higher doses and without add‐back treatment. Changes in uterine volume were more pronounced in therapies without adjuvant replacement therapy. All treatments were associated with improved quality of life scores. The significant reduction of the menstrual blood loss in women with uterine fibroids could also be confirmed in randomized controlled trials in women with heavy menstrual bleeding.^29^ Therefore, elagolix in combination with estradiol and norethindrone is approved for the treatment of fibroid‐related heavy menstrual bleeding by the US FDA.^30^

The GnRH antagonist linzagolix has been approved in the European Union for the treatment of moderate to severe symptoms of uterine fibroids in adult women of reproductive age. It is an oral medication that has to be taken once per day. Its approval is based on Phase III clinical trials (PRIMROSE trials^31^, ^32^) demonstrating significant reductions in heavy menstrual bleeding and fibroid‐related symptoms with a favorable safety profile.^33^ Linzagolix can be used with or without an add‐back therapy.

A study published in 2019 compared the antagonist relugolix at a dose of 40 mg orally each day with the agonist leuprolide at a monthly injectable dose of 1.88 mg or 3.75 mg.^34^ As findings they found that the reduction in fibroid size, uterine volume and increases in hemoglobin levels were comparable in the two groups. Relugolix was associated with an earlier effect on menstrual bleeding than leuprorelin and was generally well tolerated.

A drug containing relugolix 40 mg + estradiol 1 mg + norethisterone acetate 0.5 mg has been approved for the management of heavy menstrual bleeding associated with uterine fibroids in both the European Union and the USA.^35^, ^36^ This drug is taken orally at a similar time starting in the first 5 days of the menstrual cycle and is administered continuously until menopause. A particularly appealing aspect of this therapy is that the add‐back therapy is directly integrated into the treatment regimen. It has been shown that its use for 2 years does not induce significant bone loss. The effects of this combination therapy were examined in the LIBERTY Trials.^37^ Women had a significant reduction in symptom severity compared with those on placebo treatment. The relugolix combination therapy resulted in a substantial reduction in heavy menstrual bleeding in women with uterine fibroids and a reduction in pain as well as a reduced distress related to bleeding and pelvic discomfort.^38^

### 2.5 Progesterone receptor modulators

The other possibility of medical treatment is the use of selective progesterone receptor modulators (SPRM). The importance of progesterone in the pathogenesis of uterine fibroids explains the effectiveness of SPRM treatment. Clinical and biochemical evidence indicates that SPRMs can reduce fibroid growth and improve symptoms.^39^

A Cochrane review and meta‐analysis evaluated three SPRMs: mifepristone, ulipristal acetate, and asoprisnil.^24^ Comparing these drugs with placebo showed that SPRMs produced improvements in fibroid symptom severity and quality of life in addition to reduced menstrual blood loss and higher rates of amenorrhea. They also compared SPRMs with leuprolide acetate without finding evidence indicating a difference between the two groups for improvement in quality of life and pelvic pain. Regarding adverse effects, SPRMs were associated with endometrial changes that were benign, not related to cancer, and not precancerous.

Ulipristal acetate has been demonstrated to be an effective medical therapeutic option for heavy menstrual bleeding but is now strictly restricted as the result of some cases of serious liver injury. Before the potential liver complications associated with ulipristal acetate became known, the drug was considered a breakthrough in the treatment of uterine fibroids. An effective control of excessive bleeding due to uterine fibroids and a reduction of the fibroid size during ulipristal acetate therapy could be shown in the PEARL‐studies.^40^ Following these study results, the therapy was widely adopted worldwide until reports of adverse effects emerged. The primary complication that led to safety warnings regarding ulipristal acetate was the occurrence of serious liver injury, including cases of acute liver failure requiring liver transplantation. It was not possible to identify patients at increased risk of liver injury. Consequently, the use of ulipristal acetate became subject to stringent restrictions. The drug is now only approved under specific conditions and with regular liver function monitoring in Europe.^41^ In the USA, ulipristal acetate was not approved for fibroid treatment because of these safety concerns.

One study evaluated the impact of ulipristal acetate used 2 months before hysteroscopic myomectomy.^42^ It showed that patients who received this preoperative management had significantly shorter operative times and significantly reduced fluid absorption compared with no preoperative medical treatment (Table 2).

**TABLE 2:** Overview of the most important effects of the substances presented, including issues subjectively emphasized by the authors.

Substance	Reduction of menstrual blood loss	Reduction of fibroid volume	One major issue of concern
Tranexamic acid	✓	–	
LNG‐releasing intrauterine system	✓	–	Expulsion
Oral progestins	✓	–	
Combined oral contraceptives	✓	(✓)	Risk of thromboembolic events
GnRH agonists	✓	✓	Menopausal symptoms/bone density loss
GnRH antagonists	✓	✓	Menopausal symptoms/bone density loss
GnRH antagonist with add‐back therapy	✓	(✓?)	
Selective progesterone modulators	✓	✓	Liver injury

Abbreviations: GnRH, gonadotropin‐releasing hormone; LNG, levonorgestrel.

## 3 GLOBAL ACCESS

The majority of clinical evidence regarding medical therapies for uterine fibroids that we have presented derives from studies conducted predominantly in high‐income regions such as the USA and Europe. Experience of these therapies is limited in low‐ and middle‐income countries.

Emerging and high‐cost therapies, including GnRH antagonists like relugolix and SPRMs such as ulipristal acetate, often face regulatory and economic barriers that restrict their use in resource‐limited settings. For this reason, a comprehensive and equitable access to these substances is not available in many parts of Asia and Africa, to the best knowledge of the authors. In higher‐income Asian countries, by contrast, agents such as relugolix have been approved for the treatment of uterine fibroids. In many low‐ and middle‐income countries, more affordable agents such as oral progestins and TXA remain the mainstay of medical management despite their limited efficacy in reducing fibroid volume. Developing cost‐efficient and safe therapeutic options remains essential for achieving healthcare equity.

## 4 FUTURE OPTIONS

As uterine fibroids are such a widespread condition it is expected that there will be more research on medical treatment. After the success of relugolix and linzagolix, new therapies with GnRH antagonists might be an option. For example, to our knowledge, there is a current Phase III trial with the substance SHR7280 for persons with menorrhagia with uterine fibroids that is recruiting in Beijing, China.^43^ Furthermore, there are several studies investigating the use of vitamins and other dietary supplements as adjunctive treatments for uterine fibroids. These developments are interesting, as they may offer cost‐effective and low‐risk therapeutic alternatives in the future.

## 5 CONCLUSION

Pharmacologic treatment constitutes a key component in the management of uterine fibroids. The choice of therapeutic agents is guided by the patient's predominant symptoms and overall clinical profile. A variety of pharmacologic agents—including hormonal and non‐hormonal options—are available for the management of uterine fibroids to control symptoms and improve quality of life. However, only a limited number have received formal regulatory approval specifically for this indication. Global access to these agents is still limited and it remains a desirable goal to find more (cost‐)effective therapies.

## AUTHOR CONTRIBUTIONS

ID made substantial contributions to the conception of the work, analysis and interpretation of data for the work, drafting the work, and reviewing it critically for important intellectual content. MAL made substantial contributions to the conception and design of the work, and reviewing the work critically for important intellectual content. MZ made substantial contributions to the conception or design of the work, analysis and interpretation of data for the work, and drafting the work. All authors gave their final approval of the version to be published and agree to be accountable for all aspects of the work in ensuring that questions related to the accuracy or integrity of any part of the work are appropriately investigated and resolved.

## FUNDING

Open Access funding enabled and organized by Projekt DEAL.

## CONFLICT OF INTEREST STATEMENT

The authors have no conflicts of interest.

## Data Availability

Data sharing is not applicable to this article as no new data were created or analyzed in this study.
